# Monitoring Strategies and Intervention Policies for the Enhancement and Protection of Advanced Neuroscientific Research Post COVID-19 in Italy: Preliminary Evidence

**DOI:** 10.3389/fpubh.2021.748223

**Published:** 2021-11-25

**Authors:** Michela Balconi, Marco Bove, Maurizio Bossola, Laura Angioletti, Giulia Fronda, Davide Crivelli

**Affiliations:** ^1^International Research Center for Cognitive Applied Neuroscience (IrcCAN), Università Cattolica del Sacro Cuore, Milan, Italy; ^2^Research Unit in Affective and Social Neuroscience, Department of Psychology, Università Cattolica del Sacro Cuore, Milan, Italy; ^3^Section of Human Physiology, Department of Experimental Medicine (DIMES), University of Genoa, Genoa, Italy; ^4^Dyalisis Service, Università Cattolica del Sacro Cuore, Rome, Italy; ^5^Fondazione Policlinico Universitario A. Gemelli IRCCS, Rome, Italy

**Keywords:** neuroscience, neuropsychology, neurophysiology, healthcare, COVID-19, research management, management policies, guidelines

## R&D Activities During and After COVID-19 Pandemics

The outbreak and diffusion of COVID-19 infection had remarkably affected Research & Development (R&D) activities—which includes basic and applied research—both in a short- and long-term perspective across all European (EU) states, as well as around the globe. R&D represent a critical field of work and a strategic area of investment, with an estimated return of around 428 billion dollars [~2.5% of Gross Domestic Spending (GDP) in 2019; (1)]. Furthermore, it is well known that investment on R&D activities represent a core target in EU global development strategies. In particular, the EU Member States agreed, in the last years, to gradually increase the investments in R&D activities to the 3% of national GDP, following the so called “Barcelona target”. First exploratory analyses highlighted that the impact on research activities of the outbreak was appraised as medium or severe in 85% of reached research centers or institutions, while only 2% of them reported the absence of a relevant impact on their R&D projects (2).

Concerning the economic impact, the COVID-19 pandemic has entailed several adverse effects. In general, a negative impact has been observed on different economic sectors, as marked by the increase of unemployment rates, bankruptcy, and other financial consequences. Like the other occupational sectors, productivity in biomedical, experimental, and clinical research too has been negatively affected by the outbreak and by the related pandemics management policies due to the suspension of research activities not primarily related to COVID-19, especially for basic research institutions. Indeed, as also demonstrated by a report published by the Congressional Research Service about the effects of the COVID-19 on the federal research and development company, it emerged that the mandatory implementation of specific guidelines would have led to the interruption of the research activity carried out by many laboratories due to the loss or limited access to different equipment, the inability to purchase new instruments, and the cancellation of scientific events and conferences (3). Furthermore, in addition to the experimental research field, also the clinical research and clinical practice, due to the suspension of many routine activities, have suffered significant financial repercussions in terms of loss of wages and business, which have in turn caused several problems to the work of healthcare professionals and support personnel (4). Those phenomena produced an unprecedented crisis for global research enterprises, especially in the neuroscientific field, whose basic and clinical research activity is heavily based on first-hand data collection with test animal, test subjects or patients (5–8).

In fact, the inertia imposed by the emergency situation on neuroscience, but also neuropsychological and neurophysiological research activities, might have a too heavy price to pay in terms of social and economic aftermath. Between many, the main issues could be:

i) the delay of critical research advancements and the limited investment on developing novel and efficient applications of neuroscience and psychophysiology tools, instrumental examination, neuropsychological and neurophysiological assessment, monitoring and intervention practices to face the now renown and critical phenomenon of neuro-COVID—i.e. a clinical picture characterized by moderate-to-severe cognitive, affective, and behavioral impairments linked to COVID-19 infection (9–11);
ii) the restriction or inadequate access to clinical and research services for end-users who presents neuropsychological, neurological, and/or psychiatric symptomatology, with potentially severe consequences on their health and well-being;
iii) the direct negative effect on neuroscientific knowledge production (5) and on the development of resilience strategies for future pandemic scenarios.

The negative impact caused by the pandemics has pointed out the need to develop safe work programs, strategic rearrangement of research activities, and efficient supportive programs for economic funds (12). In particular, to limit the repercussions on scientific productivity and healthcare of the infection, some activities have been reorganized, with remote and digital tools, in most institutions and centers (13, 14). It was also asked to the scientific community to identify and implement evidence-based policies that could promote the development of new, resilient, and shared cultural practices, which involve the combination of effective remote work and on-site activities even in the neuroscientific field. Standardized and shared policies are necessary in order to improve activity and make neuroscience, neuropsychology and neurophysiology research and practice in the laboratories overcome the crisis, to capitalize present experience, and to be prepared to face potential future challenges while, at the same time, assuring public health for all the actors involved.

In line with this need, many research groups have begun to share consensus guidelines for the management of neuroscientific data collection in the pandemic period. Bikson et al. (15) proposed consensus guidelines for TMS/tES clinical services and research through the COVID-19 pandemic. In a similar work, Campanella et al. (6) reported the outcomes of a survey on the impact of COVID-19 on the use of electroencephalography (EEG) in clinical practice and research in several countries (including some EU countries such as Italy, Germany, Belgium and Czech Republic). The authors have also presented the recommendations of an international panel of experts for the safe application of EEG during and after this pandemic. Even if based on a limited number of participants and restricted to a peculiar area–i.e., electrophysiology–the study is insightful and carries within itself some precious information about situational know-how and strategies, which might reduce risks for COVID-19 spread. Among the others: a rigid check for COVID-19 symptomatology before inclusion in studies and research activities; respect of sufficient social distance and favoring of one-to-one contact, primarily between the technician and the patient, in data collection; the use of different rooms for data collection; disinfection between each recording. The authors have also suggested an update of common practices to allow safe EEG recordings in both research and clinical settings. In parallel, Sozzi et al. (7) proposed potential solutions for conducting neuropsychological assessment and neuropsychological rehabilitation with patients showing alterations of cognitive functions even during emergency situations. Furthermore, a roadmap for conducting neuroscience research in the COVID-19 era, together with the recommendations from the Society of Neuroscience in Anesthesiology and Critical Care (SNACC) Research Committee was recently published (8).

## An Applied Example: The MIRNA Project

### Aims and Project Structure

Building on such premises and on the state of the art on investment toward safe reprise of R&D activities, we will now briefly introduce an illustrative recent project that involves three main partners—the Catholic University of the Sacred Heart, the Foundation “Policlinico A. Gemelli”, and the University of Genoa—to discuss a few critical points concerning the progress of neuroscientific research in Italy during the pandemics.

The project (entitled “*Monitoring tools and intervention policies for the enhancement and protection of advanced neuroscientific research post COVID-19*”–MIRNA) was devised to evaluate and highlight the impact that the COVID-19 had on the management of basic and clinical research activities conducted by Italian laboratories for neuroscience, neurophysiology, and clinical neuropsychology during the pandemic emergency and post-emergency phases. By mapping the state of the art of such laboratories and by collecting data through a national survey, the main purposes of the present study were: (i) to define primary activities of research units and laboratories operating in the field of basic, clinical and applied neuroscience, neurophysiology and neuropsychology in Italy; (ii) to qualify and quantify critical issues resulting from the COVID-19 in those settings; (iii) to highlight the strategies used to address or mitigate those unprecedented challenges.

Firstly, to pursue such goals, Italian institutions operating within the neurophysiological, neuropsychological, and neuroscientific research fields were initially mapped in order to collect a sample as representative as possible. The systematic mapping of Italian neuroscientific, neurophysiological, and neuropsychological research facilities leads to the identification of 254 laboratories/units, which have been categorized based on location, primary research field, and category of institution.

Secondly, the outcome of such mapping, besides being used for outlining the state of the art of neuroscientific, neurophysiological and neuropsychological research institutions in Italy, has been used to define a reference population of respondents for a survey designed to identify critical issues faced by research managers and laboratory directors during the pandemic emergency and the post-emergency period and to investigate the effect of the pandemic outbreak and of related management policies on research activity and productivity, as well as the strategies and policies that have been implemented to face such issues and foster reprise of R&D activity. The survey was implemented on Qualtrics XM platform (Qualtrics LLC, Provo, UT, USA) and divided in five different parts: (i) consensus and introduction; (ii) general data on the institution and the respondent and pre-pandemic phase; (iii) research activity during Phase 1—first lockdown (from February to May 2020); (iv) research activity during Phase 2—second lockdown (October 2020 to May 2021); and (v) summary evaluations of the pandemic period (overall considerations regarding both Phase 1 and Phase 2).

### Mapping and Survey Evidence: Some First Remarks

**Figure 1:**
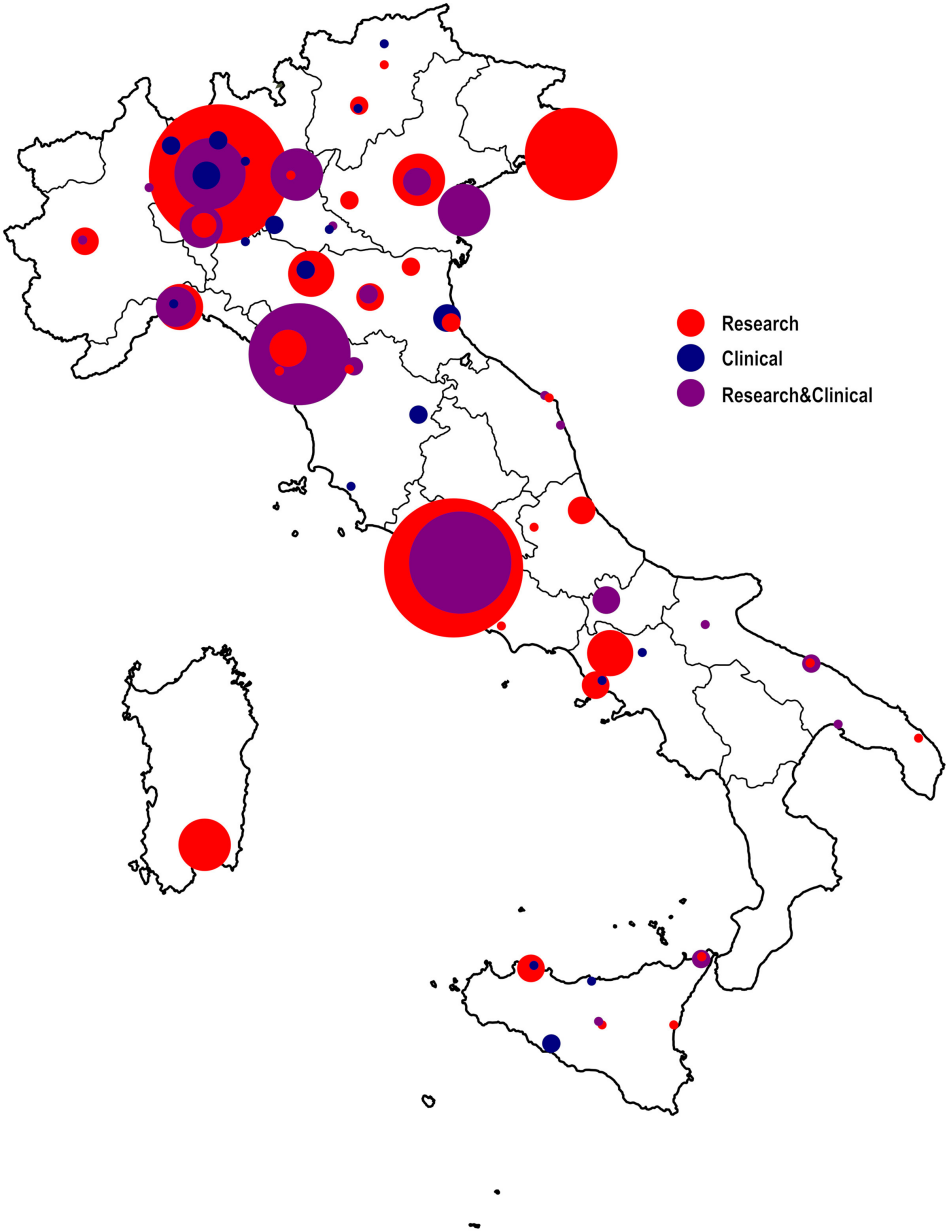
Mapping of Italian neuroscientific, neurophysiological, and neuropsychological research facilities. Laboratories and units have been clustered according to their main working activities: in red the laboratories that carry out research activities only, in blue the centers that primarily conduct clinical activities, in violet the centers that deal with both research and clinical activities. The width of the circles mirrors the number of laboratories/units in the reference geographical territory, for each clustered primary working activity (i.e., research, clinical, both).

The preliminary mapping revealed clear disparities in the regional distribution of laboratories/units involved in research and/or clinical activities in the fields of neuroscience, neurophysiology or neuropsychology (see Figure 1), with about a half of the units located in Lombardy, Lazio or Tuscany.

The whole sample of mapped institutions was constituted almost equally by purely research (45%) and mixed clinical and research (44%) units, while the institutions with a primarily clinical mission covered a smaller part of the sample (8%).

The analysis of respondents across the national territory highlighted a response rate equal to 39% (55 out of 142 laboratories/units have completed in the survey) in northern Italy, 16% (11 out of 70 laboratories/units have completed in the survey) in central Italy, and 23% (10 out of 43 laboratories/units have completed in the survey) in southern Italy.

Focusing on the sample of survey respondents, which almost equally represented primarily healthcare/clinical research professionals (53%) and primarily basic research professionals (47%), it is relevant to note that just about one fourth of them reported the existence of emergency management guidelines to help strategic decision-making and inform the rearrangement of lab/unit activities in case of a disease outbreak, a percentage that has grown up to 94% after the COVID-19 emergency. This led to a closure rate equal to 92% for purely research laboratories/units during Phase 1, compared to 52% of mixed clinical and research units and 60 % of primarily clinical units. A similar, though more restrained, scenario was observed even in Phase 2, with 44% of purely research units still closed, vs. 5% of mixed units and 10% or primarily clinical ones.

Again, another impactful observation emerging from the survey is that, while the number of submitted paper during phase 1 and 2 was almost comparable to a previous reference period (year 2019), the investigated research and clinical institutions reported a remarkable decrease of 23% for planned and submitted projects, a percentage that reaches −40% in mixed clinical-research units. We suggest that such loss of research projects in the field of basic, clinical and applied neurosciences, of their potential outcomes in terms of novel theoretical models and technological/methodological progresses, as well as of potential by-side discoveries might show its effects in the next few years.

## Conclusions

We think that the pandemic emergency that we have had to face provides, at least, the unique opportunity to reflect on the strategic value of clear, efficient and lean organizational and management guidelines, as well as of both effective vertical communication between institutions and its components and horizontal communication to share evidence-based practices between institutions. Projects like the one briefly introduced here will provide valuable food for thought concerning the development of standardized and shared practices necessary to restore pre-epidemic activities and planning, in order to ensure that R&D overcomes this crisis and potential future challenges, while also protecting the public health and all actors involved in the strategic research field of basic, clinical and applied neurosciences. Indeed, to define guidelines and new best practices for an efficient and sustainable management of these necessary activities in the short and long term is a current critical challenge, and might help containing the cost of their interruption on healthcare for the population and on individual/social well-being.

## Author Contributions

MBa, DC, LA, and GF wrote the first draft of the manuscript. All the authors contributed to the manuscript final writing and revision. All the authors read and approved the submitted version.

## Funding

This project was funded by the Italian Ministry of Education, University and Research (MIUR), with the Special Supplementary Fund for Research (in Italian: Fondo Integrativo Speciale per la Ricerca, FISR) of 2020 (FISR 2020 COVID).

## Conflict of Interest

The authors declare that the research was conducted in the absence of any commercial or financial relationships that could be construed as a potential conflict of interest.

## Publisher's Note

All claims expressed in this article are solely those of the authors and do not necessarily represent those of their affiliated organizations, or those of the publisher, the editors and the reviewers. Any product that may be evaluated in this article, or claim that may be made by its manufacturer, is not guaranteed or endorsed by the publisher.
